# Genetic Insights into Alzheimer's Disease from Cuban American Studies

**DOI:** 10.1002/alz70855_106251

**Published:** 2025-12-24

**Authors:** Larry D Adams, Jose J. Sanchez, Vanessa C. Rodriguez, Nicholas Gomez, Kara L Hamilton‐Nelson, Glenies S. Valladares, Sabrina M. Mas, Concepcion Silva‐Vergara, Pedro R Mena, Patrice G Whitehead, Michael B. Prough, Anthony J Griswold, Katalina F. McInerney, Jeffery M Vance, Michael L Cuccaro, Katrina Celis, Farid Rajabli, Margaret Pericak‐Vance

**Affiliations:** ^1^ John P. Hussman Institute for Human Genomics, University of Miami Miller School of Medicine, Miami, FL, USA; ^2^ Hussman Institute for Human Genomics, University of Miami, Miller School of Medicine, Miami, FL, USA; ^3^ Universidad Central del Caribe, Bayamón, PR, USA; ^4^ University of Miami Miller School of Medicine, Miami, FL, USA; ^5^ Dr. John T. Macdonald Foundation Department of Human Genetics, University of Miami Miller School of Medicine, Miami, FL, USA; ^6^ Department of Neurology, University of Miami Miller School of Medicine, Miami, FL, USA

## Abstract

**Background:**

Genetic studies have identified more than 80 loci associated with Alzheimer disease (AD), with APOE recognized as a major risk factor. Understanding the complete genetic landscape representation across ancestries in Alzheimer disease (AD) studies is critical to advance our ability in solving AD for everyone. Cuban Americans have a substantial European ancestral background alongside African and Amerindian ancestries. This study aims to determine whether these established AD risk loci Cuban Americans, which could enhance our understanding of the genetic architecture of AD.

**Methods:**

The CA dataset includes TOPMED imputed data from 239 individuals (119 AD, 120 cognitively unimpaired) ascertained through the Cuban American Alzheimer's Disease Initiative (CuADI). For association analysis, we employed a mixed‐model regression approach (SAIGE) where we controlled for age, gender, population substructure (first three principal components), and relatedness.

**Results:**

Association analysis in Cuban Americans replicated *SORL1* (rs74685827), *CR1* (rs6656401), *ADAM17* (rs72777026), and *RASGEF1C* (rs113706587) as risk loci for AD with odds ratios of 5.71 (*p* = 0.02), 1.70 (*p* = 0.048), 1.81 (*p* = 0.024), and 1.87 (*p* = 0.032), respectively. Conversely, *SPI1* (rs3740688) and *ADAMTS1* (rs2830489) were found as protective loci with odds ratios of 0.60 (*p* = 0.007) and 0.66 (*p* = 0.049). Importantly, the *APOE4* was significantly associated with AD (OR=2.94, *p* = 1.37×10^‐4^) showing an effect size similar to those observed in European populations.

**Conclusions:**

This study replicated the associations of *SORL1, CR1, ADAM17, RASGEF1C, SPI1*, and *ADAMTS1* with AD risk and protection are found in Cuban Americans. The large effect of the *APOE4* aligns with its established role in AD pathogenesis across different populations. These results enhance our overall understanding of the genetic underpinnings of AD and support the advancement of targeted research and therapeutic interventions.

